# Street RABV Induces the Cholinergic Anti-inflammatory Pathway in Human Monocyte-Derived Macrophages by Binding to nAChr α7

**DOI:** 10.3389/fimmu.2021.622516

**Published:** 2021-02-19

**Authors:** Carmen W. E. Embregts, Lineke Begeman, Cees J. Voesenek, Byron E. E. Martina, Marion P. G. Koopmans, Thijs Kuiken, Corine H. GeurtsvanKessel

**Affiliations:** ^1^Department of Viroscience, Erasmus Medical Center, Rotterdam, Netherlands; ^2^Independent Researcher, Rotterdam, Netherlands

**Keywords:** rabies, lyssaviruses, monocyte-derived macrophages, innate immunity, immunosuppression, cholinergic anti-inflammatory pathway

## Abstract

Rabies virus (RABV) is able to reach the central nervous system (CNS) without triggering a strong immune response, using multiple mechanisms to evade and suppress the host immune system. After infection *via* a bite or scratch from a rabid animal, RABV comes into contact with macrophages, which are the first antigen-presenting cells (APCs) that are recruited to the area and play an essential role in the onset of a specific immune response. It is poorly understood how RABV affects macrophages, and if the interaction contributes to the observed immune suppression. This study was undertaken to characterize the interactions between RABV and human monocyte-derived macrophages (MDMs). We showed that street RABV does not replicate in human MDMs. Using a recombinant trimeric RABV glycoprotein (rRABV-tG) we showed binding to the nicotinic acetylcholine receptor alpha 7 (nAChr α7) on MDMs, and confirmed the specificity using the nAChr α7 antagonist alpha-bungarotoxin (α-BTX). We found that this binding induced the cholinergic anti-inflammatory pathway (CAP), characterized by a significant decrease in tumor necrosis factor α (TNF-α) upon LPS challenge. Using confocal microscopy we found that induction of the CAP is associated with significant cytoplasmic retention of nuclear factor κB (NF-κB). Co-cultures of human MDMs exposed to street RABV and autologous T cells further revealed that the observed suppression of MDMs might affect their function as T cell activators as well, as we found a significant decrease in proliferation of CD8^+^ T cells and an increased production of the anti-inflammatory cytokine IL-10. Lastly, using flow cytometric analysis we observed a significant increase in expression of the M2-c surface marker CD163, hinting that street RABV might be able to affect macrophage polarization. Taken together, these results show that street RABV is capable of inducing an anti-inflammatory state in human macrophages, possibly affecting T cell functioning.

## Introduction

Rabies is a zoonotic viral encephalitis responsible for 60,000 reported human deaths annually, although the true burden is suspected to be much higher (1). The disease is caused by members of the genus *Lyssavirus*, -ssRNA viruses of the *Rhabdoviridae* family. While multiple lyssaviruses have been reported to cause rabies in humans, over 99 percent of all reported human cases are caused by dogs infected with rabies virus (RABV) (2). Nevertheless, in North and South America bats are the main source of human infections, mostly caused by silver-haired bat rabies virus (SHBRV) (3).

While effective pre- and post-exposure prophylaxis is available, no treatment options are currently available once clinical symptoms occur. This makes rabies the deadliest zoonosis with a case-fatality rate approaching 100%. The virus is able to reach the CNS without triggering a strong local immune response (4, 5); neither is a systemic immune response induced, as indicated by the low or absent neutralizing antibodies at the end-stage of disease of most patients (6, 7). This lack of immune activation is caused by limited viral replication at the inoculation site (8, 9), as well as by active immune evasion and suppression, as extensively reviewed (10, 11). Previous research identified major immune evasive mechanisms, including the blocking of RIG-I activation by the nucleoprotein (N) (12–14) and the IFN signal transduction pathway by the phosphoprotein (P) (15–17).

To date, the majority of research on immunosuppressive mechanisms focused on the response in neurons and the CNS. While the absence of an effective local immune response after infection with RABV has been described (4), it is not clear how exactly RABV affects local immune cells at the site of infection. Suppression of local immune cells may be responsible for the lack of immune response in later stages of diseases, hence increasing the knowledge on interactions between RABV and local immune cells is indispensable in the development of effective and targeted treatment strategies. Given this importance, we aimed to investigate the functional effects of street RABV on macrophages. Macrophages are versatile innate immune cells and play an essential role in the uptake and clearance of pathogens, and initiation of a strong local response by producing pro-inflammatory cytokines, chemokines and nitric oxide (NO) (18, 19). After being bitten or scratched by a rabid animal, RABV first encounters resident macrophages in skin and muscle tissue, after which the inflicted physical damage and the presence of bacteria and other microbes in the wound area induce a strong influx of immune cells (20, 21). Large numbers of infiltrated macrophages have been found in peripheral sites after inoculation with RABV as well (22); however, the interactions between RABV and macrophages have been studied incompletely. The few studies focusing on the direct effects of RABV on macrophages showed that various RABV strains were able to induce NO production and CXCL10 expression in RAW264 murine macrophages (23), and induced macrophage apoptosis through involvement of various caspases (24). In the aforementioned papers, lab-adapted or attenuated RABV strains and murine macrophage-like cell lines were used, therefore the effects of street RABV strains on primary macrophages remain unstudied.

Macrophages have a high functional plasticity. Upon encountering pathogens, and in combination with cytokines produced by resident cells, macrophages polarize toward diverse and distinct functional phenotypes, as are normally grouped into classically activated or inflammatory M1 phenotypes, and alternatively activated or anti-inflammatory M2 phenotypes (25). A typical type 1/Th1 immune response, associated with M1 macrophages, aids the elimination of intracellular pathogens while a typical type 2/Th2 response, associated with M2 macrophages, will reduce viral clearance. Shifting macrophage polarization toward a phenotype beneficial for the virus is a strategy known for many viruses, as extensively reviewed (26). Studies on the effects of street RABV strains on macrophage polarization are lacking.

Nicotinic acetylcholine receptors (nAChr) are a family of ligand-gated pentameric ion channels with a variety of pharmacological functions. The nAChr α7 is a homopentameric nAChr that consists of five α7 subunits and is involved in the cholinergic anti-inflammatory pathway (CAP), characterized by a decreased inflammatory response of macrophages upon binding of a typical ligand (nicotine, acetylcholine) to nAChr α7 (27). RABV glycoprotein (RABV-G) can potentially bind to many nAChrs including nAChr α7, but functional effects of this binding have not yet been studied (28). We hypothesize that binding to the nAChr α7 leads to suppression of the inflammatory response of macrophages.

In the present study we showed that a street RABV strain does not replicate in human monocyte-derived macrophages (MDMs). We confirmed specific binding of RABV-G to nAChr α7 on human MDMs and, for the first time, showed that a viral protein (RABV-G) was able to induce the cholinergic anti-inflammatory pathway (CAP) in MDMs. We found that the induction of the CAP was related to cytoplasmic retention of NF-κB in RABV-exposed MDMs, and that exposure of MDMs with RABV decreased proliferation of autologous T cells and increased IL-10 production *in vitro*. In parallel, exposure of MDMs to RABV did not induce typical M1 phenotypic markers, but instead upregulated the M2-c phenotypic marker CD163, hinting at the possibility that RABV is able to shift macrophage polarization toward an anti-inflammatory M2-like phenotype.

## Methods

### Virus

A street rabies virus (RABV) strain known for its capability to cause human infections in North America, the silver-haired bat rabies virus (SHBRV), was used. The virus was propagated in the human neuroblastoma cell line SK-N-SH in Eagle's Minimum Essential Medium (EMEM) with Earle's Balanced Salt Solution (EBSS) (Lonza), supplemented with 10% (v/v) Fetal Calf Serum (FCS), 100 U penicillin (Gibco), 100 mg/mL streptomycin (Gibco), 2 mM L-glutamine (Gibco), 1% non-essential amino acids (Lonza), 1 mM sodium pyruvate (Gibco) and 1.5 mg/mL sodium bicarbonate (Gibco). Virus titrations were performed by the median tissue culture infective dose (TCID_50_) endpoint dilution method of Reed and Muench (29) using the mouse neuroblastoma cell line MNA. MNA cells were cultured in Dulbecco's Modified Eagle Medium (DMEM, Gibco) supplemented with 10% FCS, 100 U penicillin, 100 mg/mL streptomycin, 2 mM L-glutamine and 1 mM sodium pyruvate (Gibco). SK-N-SH and MNA Cells were maintained at 37°C with 5% CO_2_.

### Primary Cell Isolation and Macrophage Maturation

Peripheral blood mononuclear cells (PBMCs) were isolated from buffy coats obtained from healthy, non-smoking and non-rabies vaccinated blood donors (Sanquin). Written informed consent for research use of donated blood was obtained by the Sanquin blood bank. Peripheral blood mononuclear cells were obtained by density centrifugation using Ficoll Paque PLUS (GE Healthcare). Monocytes and T cells were obtained from PBMC fractions by magnetic associated cell sorting using CD14^+^ and CD3^+^ beads, respectively, following manufacturers guidelines (Miltenyi Biotec). Purity of the sortings was confirmed by flow cytometry using a BD Lyric flow cytometer (BD Biosciences).

Monocytes were seeded at a density of 100,000 cells per well in 96-well plates and were maturated for 6 days in Roswel Park Memorial Institute-1640 (RPMI-1640) medium containing 10% pooled human serum (Sanquin), 1% (v/v) GlutaMAX (Gibco), and 20 ng/mL monocyte colony-stimulating factor (M-CSF, R&D Systems). Cells were maintained at 37°C with 5% CO_2_ and the medium was replaced on day 2 and day 4.

### Infection of Human Monocyte-Derived Macrophages

Mature human monocyte-derived macrophages from *n* = 6 individual donors, obtained on day 6 of culture, were exposed to RABV for 1 h in serum-free medium using a MOI of 0.1, 1, 10, or 50, after which the cells were washed once and were incubated in complete medium. At 8, 24, 48, and 72 h post-infection, cell culture supernatants were harvested for virus titration. Additionally, mature macrophages from *n* = 2 individual donors were exposed to RABV using a MOI of 10 and 50, as described above, and supernatant was collected at 72 h post-infection. Cells were fixed in 80% acetone for immunofluorescent detection of the nucleoprotein using a mouse FITC-labeled RABV-N antibody (Fujirebio). Nuclear counterstaining with Hoechst 33342 (Sigma Aldrich) was included before image acquisition using a Zeiss LSM700 confocal laser scanning microscope.

Virus titrations were performed by the TCID_50_ endpoint dilutions method of Reed and Muench using the mouse neuroblastoma cell line MNA, as described before. A total of six donors were included for the MOIs 0.1 and 1, and two donors for the MOIs of 10 and 50. Two wells were infected for every condition and all supernatants were titrated in triplicate. Infection of the highly susceptible neuroblastoma cell lines SK-N-SH and MNA cells were included as positive controls for virus infectiousness.

### rRABV-tG Binding Assay

The ability of the RABV glycoprotein to bind to human monocyte-derived macrophages was investigated using a recombinantly expressed trimeric form of RABV-G, based on the sequence of the Pasteur strain RABV [rRABV-tG, described in (30)]. rRABV-tG was labeled with a 0.1 mg/mL solution of FITC (Sigma Aldrich) in 0.5 M bicarbonate buffer (pH 9.5) for 1 h under constant stirring, after which unbound FITC was removed by overnight dialysis in PBS. Mature macrophages were incubated with 50 μg/mL FITC-labeled rRABV-tG for 30 min at 4°C. In parallel, macrophages were incubated with ATTO-633 conjugated alpha-bungarotoxin (α-BTX; Alomone labs) or with an antibody against the nicotinic acetylcholine receptor α7 (nAChr α7; Alomone Labs) followed by an Alexa594 goat-anti-rabbit conjugate (Invitrogen). Nuclear counterstain with Hoechst 33342 (Sigma) was included before cells were imaged using a Zeiss LSM700 confocal laser scanning microscope.

For quantification of rRABV-tG binding, mature macrophages (*n* = 6 individual donors) were detached using Accutase (Merck Millipore), blocked with 10% pooled human serum in PBS for 30 min and incubated with various concentrations of rRABV-tG (0–100 μg/mL) for 30 min at 4°C. To verify the binding of rRABV-tG to the nAChr-α7, macrophages were incubated with various concentrations of the nAChr α7 antagonist alpha-bungarotoxin (α-BTX, 0-50 ug/mL) for 15 min before addition of rRABV-tG. After two washes in FACS buffer, binding of rRABV-tG, as well as the expression of nAChr α7 was quantified using a BD Lyric flow cytometer (BD Biosciences). Data were analyzed using FlowJo V10.6.2.

### Macrophage Stimulation and TNF-α Cytokine Assay

The anti-inflammatory effect of RABV on macrophages was studied using a lipopolysaccharide (LPS) stimulation. LPS is an outer membrane component of Gram-negative bacteria and is known to induce a strong inflammatory response. Briefly, mature macrophages were exposed to RABV (MOI of 10, 25, or 50) for 1 h and subsequently with LPS (100 ng/mL) for 6 h. Treatment with acetylcholine (1 μg/mL) was used as a positive control for induction of the cholinergic anti-inflammatory pathway. Wells without LPS challenge, or LPS challenge without pre-exposure to infectious RABV or acetylcholine, were included as negative and positive control, respectively. To confirm the binding of RABV to nAChr α7, the specific antagonist alpha-bungarotoxin (α-BTX, 2 μg/mL) was used as a pre-treatment before cells were exposed to RABV or acetylcholine, and subsequently to LPS (**Figure 3A**). All conditions were tested in mature macrophages from six individual donors, except the highest dose of RABV (MOI of 50, with and without pre-treatment of α-BTX or acetylcholine) which were tested in mature macrophages from a total of nine donors. All conditions were tested in triplicate wells.

Supernatants were collected after 6 h of stimulation with LPS and TNF-α concentrations were determined using the Legendplex TNF-α cytometric bead assay (BioLegend). Bead analysis was performed by a BD FACS Lyric flow cytometer and the data was analyzed using FlowJo V10.6.2. Relative TNF-α reduction per individual donor was obtained by normalizing MFI's against the TNF-α production of the corresponding wells stimulated with LPS only.

### Quantification of NF-κB Nuclear Translocation and Retention

Macrophages (*n* = 6 donors) were exposed to RABV (MOI of 50) or acetylcholine (1 μg/mL) and afterwards challenged for 6 h with LPS (100 ng/mL) as described above. Wells were washed twice with PBS, fixed with 4% paraformaldehyde (PFA) in PBS for 15 min and permeabilized using PBS 0.1% Triton X-100 for 15 min. After blocking for 30 min with 2% FCS in PBS, wells were stained with a monoclonal antibody against the NF-κB the p65 subunit (Santa Cruz) and goat-anti-mouse Alexa488 (Invitrogen). Nuclei were counterstained with Hoechst 33342 (Sigma Aldrich) and wells were imaged using a Zeiss LSM700 confocal laser scanning microscope.

An ImageJ macro was designed to perform automatic and user-independent quantification of nuclear and cytoplasmic NF-κB. Briefly, z-stacks were projected into single images by summing the intensity of all slices. Nuclear regions were defined by the Hoechst 33342 staining and a binary mask was created using an intensity threshold. Total NF-κB was detected by applying the Li intensity threshold on the Alexa488 channel, after which the signal was converted into a binary mask. In order to obtain the cytoplasmic NF-κB, the binary mask containing the nuclei was subtracted from the total NF-κB channel. The area and mean NF-κB intensity were measured in the original z-projection. Lastly, total NF-κB expression was determined by area ^*^ intensity of both the nuclear and cytoplasmic region and the average nuclear/cytoplasmic ratios were determined for each image. At least three high-magnification fields were imaged per well, and nuclear/cytoplasmic ratios were determined for six donors in total.

### T Cell Co-culture

Macrophages (*n* = 7 donors) treated with RABV (MOI of 50) for 1 h were washed twice with medium (RPMI supplemented with 10% FCS, 100 U penicillin, 100 mg/mL streptomycin and 2 mM L-glutamine) before autologous T cells were added. CD3^+^ T cells were stained with 2 μM carboxyfluorescein succinimidyl ester (CFSE) as described before (31), were activated with 2.5 μg/mL α-CD3 (Invitrogen) and α-CD28 (Invitrogen) and were co-cultured with macrophages in a 1:1 ratio. Non-stimulated T cells, as well as stimulated T cells cultured without macrophages, were taken along as controls for each donor. Cells were cultured in 3.5 days after which proliferation was determined by flow cytometry. To this end, T cells were washed twice with PBS, stained with the fixable viability dye ZombieViolet (Biolegend), fixed with 4% PFA for 15 min and subsequently stained for 30 min with anti-CD4-APC and anti-CD8-BV605 (Biolegend) in FACS buffer. Flow cytometry analysis was performed using a BD FACS Lyric flow cytometer and the data were analyzed using FlowJo V10.6.2. Cell culture supernatant at the moment of T cell harvest (*n* = 6) was stored at −80°C and was used for quantification of cytokines using the Legendplex cytometric bead assay (Biolegend) as described above.

### Macrophage Maturation and Polarization

Mature macrophages (*n* = 6 donors) were stimulated for 48 h with complete medium containing IFN-γ (20 ng/mL, R&D Systems) and LPS (100 ng/mL, Sigma Aldrich), with IL-4 (20 ng/mL, R&D Systems), or with IL-10 (20 ng/mL R&D Systems), to induce the M1, M2a, or M2c phenotype, respectively. To investigate the effect of rabies virus on macrophage polarization, macrophages were stimulated with complete medium containing RABV (MOI of 10). Expression of macrophage phenotypical markers was investigated by flow cytometry. Non-polarized macrophages, cultured for 48 h with complete medium without additional cytokines, were taken along as controls for each donor.

Macrophages were dissociated from the wells using Accutase (Merck Millipore) and were washed twice with PBS before staining for 30 min with the fixable viability dye ZombieViolet (Biolegend). Cells were fixed with 4% PFA for 15 min, and after Fc receptor blocking with Human TruStain FcX (Biolegend), cells were stained with the following antibodies in FACS buffer (PBS with 2% fetal calf serum, 0.2 mM EDTA, 0.01% sodium azide): anti-CD80-FITC, anti-HLA-DR-APC-Cy7, anti-PD-L1-APC, anti-CD163-PE, anti-CD200R-PE-Cy7, and anti-CD206 (BV786) (All Biolegend). Mean fluorescent intensities were quantified by flow cytometry using a BD Lyric flow cytometer (BD Biosciences) and the data were analyzed using FlowJo V10.6.2. Cell culture supernatant at 48 h post-stimulation (*n* = 4) was stored at −80°C and was used for quantification of cytokines using the Legendplex cytometric bead assay (Biolegend) as described above.

### Statistical Analysis

All statistical analyses were performed using SPSS Statistics 25 (IBM). For the rRABV-tG binding assay, the cytokine assays and the polarization study, One-Way ANOVAs were performed followed by Tukey's *post-hoc*-tests to test significant differences between treatments. For the NF-κB assay and the proliferation assay, paired sample *T*-tests were performed to determine significant differences. *P*-values < 0.05 were considered significant and all results were expressed as mean ± SEM.

## Results

### Street RABV Does Not Replicate in Human Monocyte-Derived Macrophages

**Figure 1:**
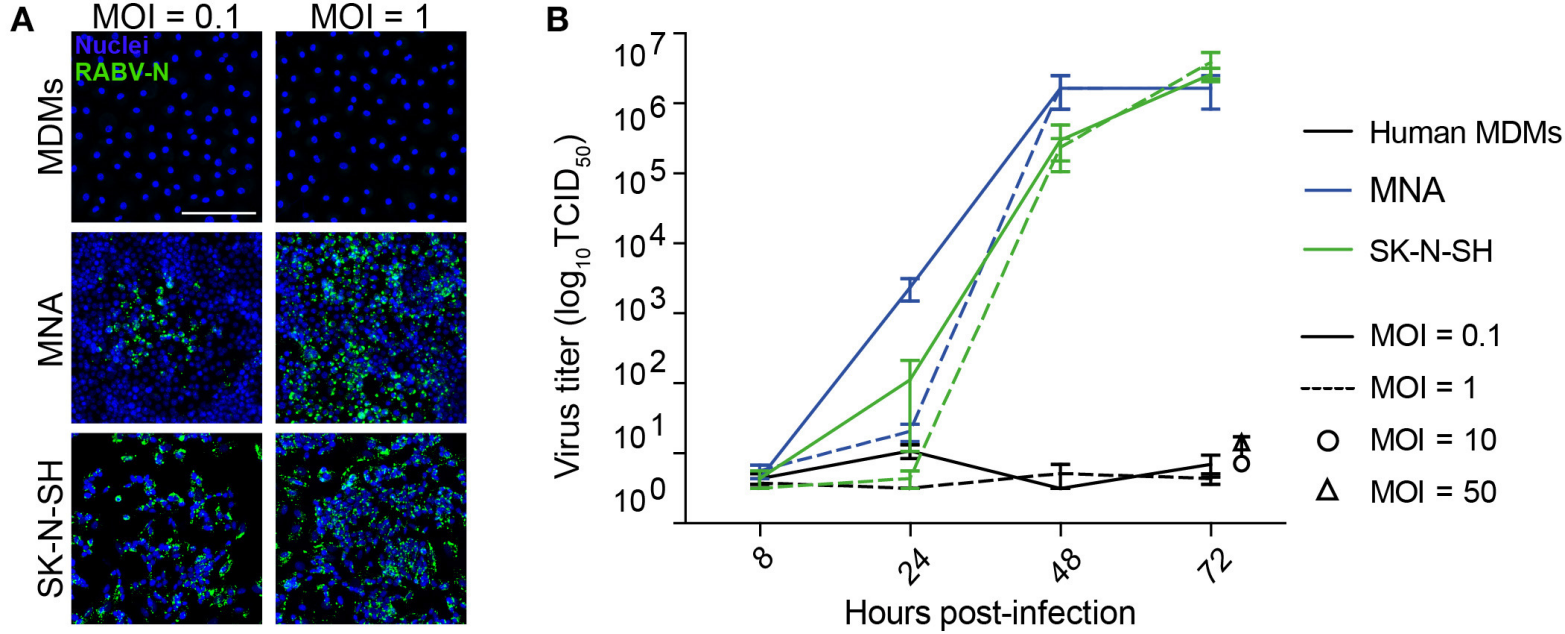
Qualitative and quantitative assessment of the ability of RABV to infect mature human monocyte-derived macrophages. Human monocyte-derived macrophages (MDMs) were exposed to street RABV at a MOI of 0.1, 1, 10 or 50, and at 72 h post-infection (hpi) cells were fixed and stained with an antibody against the RABV-N protein **(A)**. The white scalebar represents 500 μM and all images were acquired at the same magnification. In parallel, supernatants of 8, 24, 48, and 72 hpi (MOI 0.1 and 1; *n* = 6) or 72 hpi (MOI 10 and 50; *n* = 2) were titrated on MNA cells **(B)**. Bars represent the mean ± SEM of two-six donors; for each condition, two wells were infected per condition and all supernatants were titrated in triplicate. Highly susceptible MNA and SK-N-SH cells were taken along as positive controls.

To determine the immunomodulatory effects of RABV-macrophage interactions, we first investigated if exposure to street RABV leads to a productive infection in human MDMs, measured by an increase in virus over time. To this end, mature MDMs were exposed to street RABV in a MOI of 0.1, 1, 10 or 50, and at 8, 24, 48, and 72 h post-infection (hpi) the presence of virus was examined by fluorescent microscopy and virus titration. The highly susceptible human neuroblastoma cell lines MNA and SK-N-SH were taken along as positive controls. RABV-N staining was detected from 24 hpi onwards in the MNA and SK-N-SH cells, but remained absent throughout all time-points in the human MDMs (Figure 1A). MNA and SK-N-SH cells exposed to a MOI of 1 reached a plateau phase around 48 hpi, with titers between 10^6.17^ and 10^6.50^ TCID_50_/mL, and cells inoculated with a MOI of 0.1 reached this phase around 72 hpi (Figure 1B). While a low positive signal (10^1^ TCID_50_/mL) was detected at 24 hpi in human MDMs that were exposed to a MOI of 1, no increase was observed at 48 and 72 hpi, or in MDMs infected with a higher MOI. This lack of increase of present virus, as well as the absence of RABV-N protein at all tested time points, shows that inoculation with a street strain of RABV does not lead to a productive infection of mature human MDMs.

### RABV Glycoprotein Binds to nAChr α7 on Human Monocyte-Derived Macrophages

**Figure 2:**
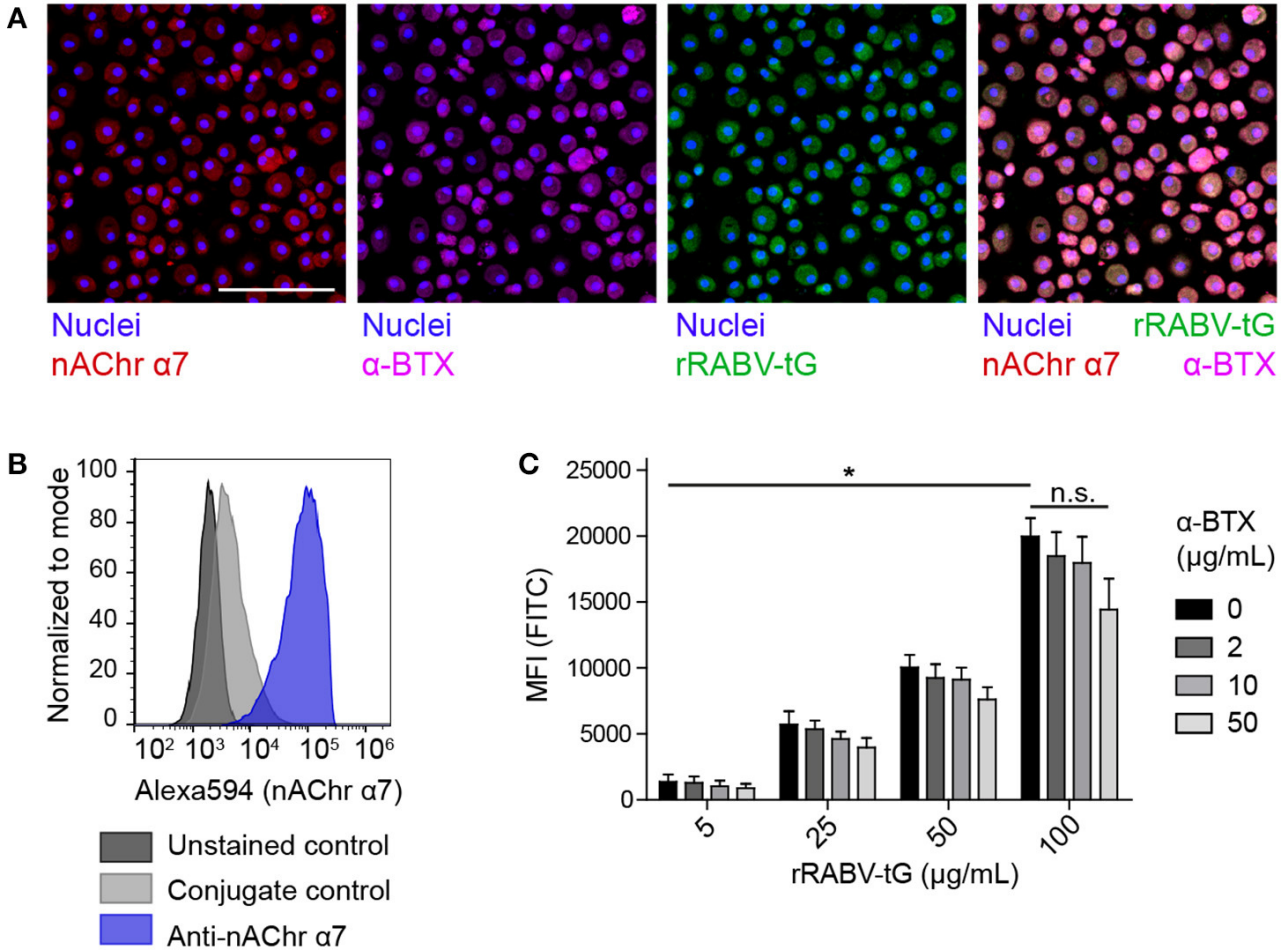
Presence of nAChr α7 on mature human monocyte-derived macrophages and binding of recombinant trimeric RABV glycoprotein to this receptor. **(A)** The presence of nAChr α7 on mature human MDMs, as well as the binding of α-BTX and FITC-labeled rRABV-tG was visualized by confocal laser scanning microscopy. Nuclei were stained with Hoechst 33342. The white scalebar represents 500 μM. The lower right plot shows an overlaid image of the nAChr α7 staining and α-BTX and rRABV-tG binding. Presence of nAChr α7 was confirmed by flow cytometry **(B)** and this technique was also used to quantify binding of FITC-labeled rRABV-tG, shown by the mean-fluorescent intensity (MFI) of the FITC channel representing rRABV-tG binding, as well as the blocking of binding by pre-incubation with the nAChr α7 specific antagonist α-BTX **(C)**. Bars represent the mean ± SEM for a total of six donors. *P*-values < 0.05 were considered significant and are indicated with an asterisk (*). Non-significant comparisons were indicated with n.s.

As a first step in testing if RABV is able to induce the cholinergic anti-inflammatory pathway (CAP) in human macrophages, we investigated the ability of RABV-G to bind to nicotine acetylcholine receptor alpha-7 subunit (nAChr α7) on human MDMs. Binding was examined by both flow cytometry and confocal microscopy, using FITC-labeled recombinant trimeric RABV-G (rRABV-G). First, expression of nAChr α7 was examined on mature MDMs by confocal microscopy (Figure 2A) and flow cytometry (Figure 2B), confirming a strong expression of this receptor. In parallel, investigation on the binding of nAChr α7 antagonist alpha-bungarotoxin (α-BTX) and the FITC-labeled rRABV-tG by confocal microscopy, showed that both α-BTX and rRABV-tG could efficiently bind to human MDMs (Figure 2A). Next, macrophages were incubated with various concentrations of rRABV-tG, and flow cytometric analysis revealed a concentration-dependent binding to the MDM surface (Figure 2C). In order to confirm that rRABV-tG binds to nAChr α7, a pre-treatment with α-BTX was used. Although no significant inhibition of rRABV-tG binding was observed at any of the tested α-BTX concentrations (0–50 μg/mL), binding showed a decreasing trend with increasing concentrations of α-BTX (Figure 2C). This confirms that α-BTX and rRABV-tG compete for the same receptor and that indeed rRABV-tG binds to nAChr α7 on human MDMs.

### Binding of RABV to nAChr α7 Induces the Cholinergic Anti-inflammatory Pathway in Human Macrophages Through Cytoplasmic Retention of NF-κB

**Figure 3:**
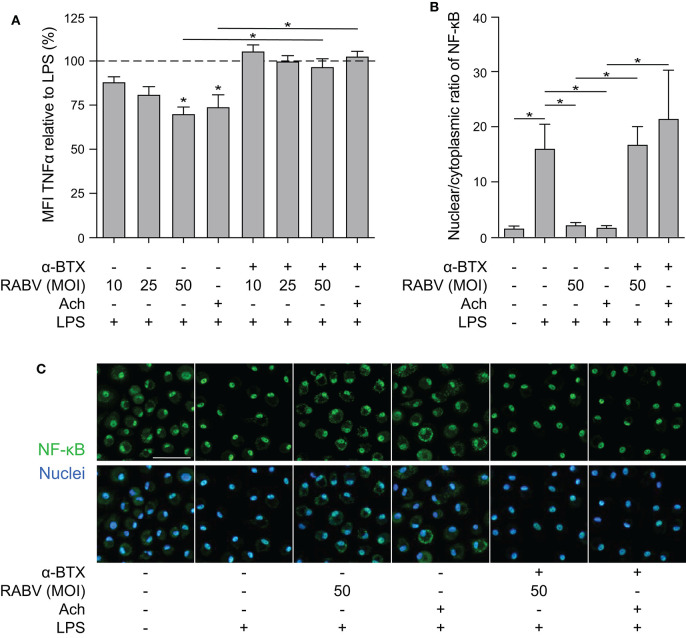
Quantification of the anti-inflammatory effect induced of RABV on human macrophages and the role of cytoplasmic retention of NF-κB. **(A)** TNF-α production in human macrophages after 6 h stimulation with LPS (100 ng/mL), with (“+”) or without (“–”) pre-treatment of α-BTX (2 μg/mL), RABV or acetylcholine (Ach; 1 μM), or a combination. Bars represent the mean ± SEM of TNF-α normalized to cells stimulated with LPS only (positive control, dotted horizontal line) for each individual donor (*n* = 6–9). Horizontal lines represent paired comparisons, whereas asterisks without horizontal lines indicate comparisons with the positive control (LPS only). **(B)** Nuclear and cytoplasmic NF-κB was quantified by image analysis in ImageJ using a batch image analysis. Bars represent the mean ± SEM of six donors, for which three high-magnification fields were analyzed per treatment. *P*-values < 0.05 were considered significant and are indicated with an asterisk (*). Representative pictures of NF-κB staining are depicted in panel **(C)**. Pictures where the NF-κB staining (green) completely overlaps with the nuclei (blue) show full nuclear translocation, whereas pictures with non-complete overlap of NF-κB and nuclei indicate cytoplasmic retention of NF-κB. The white scalebar represents 500 μM and all images were acquired at the same magnification.

After confirming that rRABV-tG binds to nAChr α7 on human macrophages we set out to investigate whether this binding could result in induction of the cholinergic anti-inflammatory pathway (CAP). Mature MDMs were exposed to various doses of RABV (MOI = 10, 25, 50), after which they were stimulated with LPS for 6 h. Quantification of TNF-α by cytometric bead assays showed that exposure to high concentrations of RABV (MOI of 50) resulted in a significant decrease in TNF-α upon stimulation with LPS (Figure 3A). The decrease in TNF-α production (30.1% on average) was similar to the decrease observed with wells pre-treated with acetylcholine, a prototypical ligand of nAChr α7. The lower concentrations of RABV (MOI of 10 and 25) did not induce this decrease, indicating that higher viral concentrations were required to induce the CAP. Importantly, by pre-treating macrophages with α-BTX we were able to inhibit the induction of the CAP, as TNF-α production was similar to the controls treated with LPS only. This confirms that observed induction of the CAP is caused by specific binding of RABV-G to nAChr α7.

Next, we aimed at investigating whether induction of the CAP by RABV also involves cytoplasmic retention of NF-κB, as is described for the prototypical ligands nicotine and acetylcholine. Macrophages were either treated with RABV (MOI of 50) or acetylcholine before challenge with LPS, after which the cells were fixed and stained with an antibody against the NF-κB p65 subunit. Nuclear and cytoplasmic NF-κB were quantified in confocal microscopy images using ImageJ batch image processing. Exposure of human MDMs to LPS led to nuclear translocation of NF-κB, hereby significantly increasing the nuclear/cytoplasmic ratio (Figures 3B,C). This translocation could be blocked almost completely by pre-treating macrophages with RABV or acetylcholine, causing cytoplasmic retention of NF-κB. Notably, the cytoplasmic retention caused by both RABV and acetylcholine could be blocked by pre-treating the cells with the nAChr α7 specific antagonist α-BTX, confirming that binding of RABV to the nAChr α7 is essential for the observed NF-κB cytoplasmic retention.

### RABV-Exposed Macrophages Suppress T Cell Proliferation and Increase IL-10 Production

**Figure 4:**
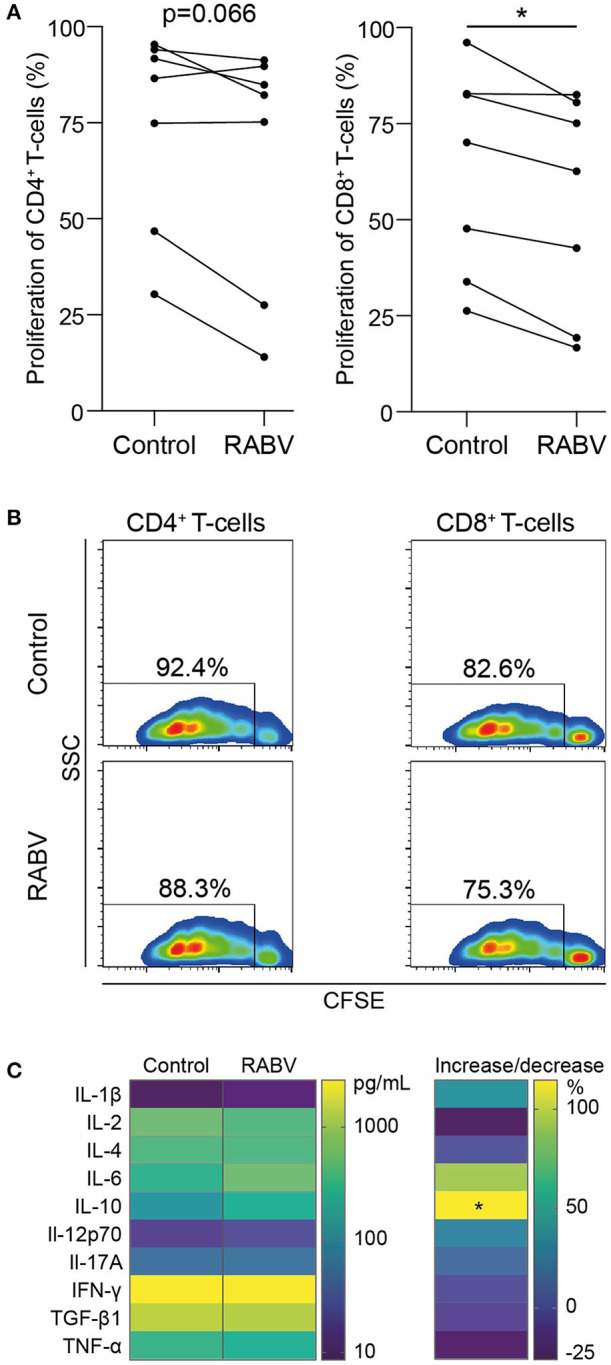
Proliferation and cytokine production of autologous T cells during *in vitro* co-culture with human monocyte-derived macrophages exposed to RABV. Proliferation of CFSE-stained T cells was quantified by flow cytometry after 3.5 days of co-culture with autologous MDMs previously exposed to RABV (MOI = 50). T cells cultured in the presence of macrophages that were not exposed to RABV were taken along as controls. Individual donors (*n* = 7) are shown in **(A)** and representative CFSE plots showing the fluorescent intensity and percentages of proliferation are shown in **(B)**. Cytokine concentrations were determined in the supernatant (*n* = 6) using a cytometric bead assay and are shown in **(C)**. The left panel shows mean absolute values in pg/mL, the right panel shows the percentage of increase or decrease in cytokine production in the RABV-exposed co-culture when compared to the control co-culture. *P*-values < 0.05 were considered significant and are indicated with an asterisk (*).

Macrophages play an important role in initiating adaptive immune responses by presenting antigens and producing various cytokines that either inhibit or activate T proliferation upon interaction with these cells. We investigated if the observed anti-inflammatory response of RABV on human MDMs could also affect T cells *in vitro*, be decreasing proliferation or by changing the cytokine profile. To study this, we performed co-cultures of macrophages exposed to RABV (MOI of 50), and autologous T cells that had been activated with soluble α-CD3 and α-CD28. T cells were stained with CFSE, and after 3.5 days T cell proliferation was analyzed by flow cytometry. Proliferation of CD8^+^ T cells was significantly decreased upon co-culture with RABV-exposed macrophages (average decrease of 8.6%) when compared to T cells cultured with control macrophages not exposed to RABV (Figures 4A,B). Although not significant, CD4^+^ T cells showed a similar trend (average decrease of 7.8%). This shows that exposure of human MDMs to RABV is able to suppress T cell proliferation *in vitro*. In addition, quantification of cytokines revealed a significant increase in IL-10 in the supernatant of the co-culture with RABV-exposed macrophages (253.59 pg/mL on average), compared to the control co-culture (118.49 pg/mL on average) (Figure 4C). The other cytokines did not differ significantly. However, the increase in IL-10 shows that besides decreasing proliferation, RABV-exposed macrophages are able to affect T cell functioning by altering cytokine production.

### RABV Does Not Polarize Human Macrophages Toward a Typical M1 or M2 Phenotype

**Figure 5:**
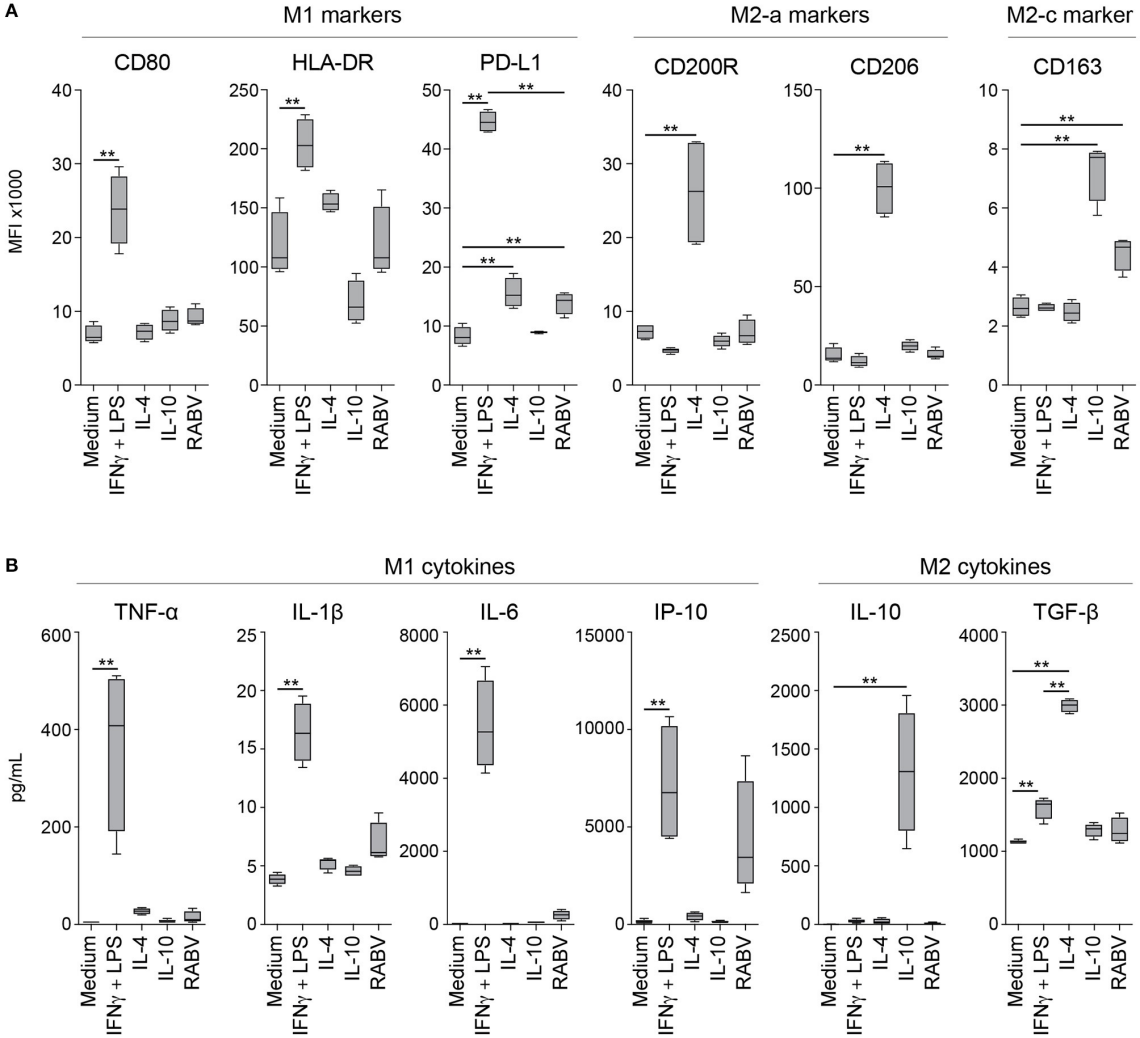
Expression levels of macrophage phenotypical markers and produced cytokines after 48 hours of polarization with various cytokines or RABV. Panels represent typical M1 markers (CD80, HLA-DR and PD-L1), M2-a markers (CD200R and CD206) and the M2-c marker CD163 **(A)** or M1 (TNF-α, IL-1β, IL-6, and IP-10) or M2 cytokines (IL-10 and TGF-β) **(B)**. Mean fluorescent intensities (MFI) were measured by flow cytometry after 48 h of polarization with LPS and IFN-γ (M1), IL-4 (M2a), IL-10 (M2c), or RABV (MOI = 10), cytokines were quantified in the same samples using a cytometric bead assy. Bars represent the mean ± SEM of *n* = 6 (phenotypical markers) or *n* = 4 (cytokines) individual donors. Values were considered significant when *P* < 0.05. *P* < 0.005 are indicated with (**).

After showing that exposure to RABV induces an anti-inflammatory pathway in human MDMs, we investigated whether longer exposure (48 h) to RABV (MOI of 10) was able to induce polarization of human MDMs. A panel of M1, M2-a, and M2-c phenotypical markers was used to investigate the macrophages' phenotype by flow cytometry; polarizing cytokine cocktails known to induce M1, M2-a and M2-c phenotypes were taken along as controls. Additionally, the production of prototypical M1 (TNF-α, IL-1β, IL-6, and IP-10) and M2 (IL-10 and TGF-β) was quantified in the cell culture supernatant of the polarized macrophages. Polarization with IFN-γ and LPS induced a typical M1 phenotype, characterized by significant upregulation of the surface markers CD80, HLA-DR, and PD-L1 (Figure 5A) and an increased production of the M1-cytokines TNF-α, IL-1β, IL-6, and IP-10 (Figure 5B). IL-β and IP-10 were also produced by RABV-stimulated macrophages, however, the levels were not significantly different from the medium controls. The production of the M2-cytokine TGF-β was significantly induced, but levels were significantly lower when compared to macrophages stimulated with IL-4. In contrast, the typical M2-a markers CD200R and CD206 were upregulated after polarization with IL-4. CD163, a typical M2-c marker, was not only upregulated after polarization with IL-10, but also after exposure to RABV. Although PD-L1 was also upregulated in macrophages polarized in the presence of RABV, the upregulation was lower than observed in macrophages polarized with IL-4 or with IFN- γ and LPS. The M2-cytokine IL-10 was only detected in the supernatant of IL-10-stimulated macrophages. While the detected level most likely reflects the input cytokines used for stimulation, it does show that no IL-10 is produced in macrophages polarized with different stimuli. All together these findings demonstrate that while RABV does not induce a typical M1 or M2 phenotype, it does affect the macrophage polarization phenotype.

## Discussion

In this study we investigated if street RABV can induce anti-inflammatory pathways in human MDMs and if interaction between RABV and macrophages can affect T cell proliferation. Knowledge on the early interactions between RABV and local immune cells are of utmost importance in order to develop and improve efficient post-exposure treatments. We demonstrate that a street RABV strain (SHBRV) has an anti-inflammatory effect on human MDMs by inducing the CAP through binding of RABV-G to nAChr α7, characterized by cytoplasmic retention of NF-κB and a decreased TNF-α response upon LPS stimulation. We further show that exposure of macrophages to RABV decreases T cell proliferation *in vitro*, while at the same time increasing production of the anti-inflammatory cytokine IL-10. Lastly, extended exposure of 48 h upregulates surface expression of CD163, a marker of the anti-inflammatory M2-c phenotype.

Before studying the immunomodulatory effects of street RABV on human MDMs, we investigated if street RABV is able to replicate in human MDMs and concluded that this is not the case (Figure 1). This lack of replication was also reported in murine bone marrow- or peritoneal-derived macrophages infected with various street RABV strains (32), indicating that primary macrophages are non-permissive to infection with street RABV strains. In contrast, primary murine macrophages did show replication of the lab-adapted CVS-11 and attenuated HEP Flury strains (33) and the recombinant matrix gene-deleted vaccine strain rRABV-ΔM (34). This indicates that in contrast to street RABV strains, some attenuated strains are able to productively infect primary macrophages.

We next confirmed that RABV-G specifically binds to the nAChr α7 on human MDMs, as binding of the recombinant trimeric rRABV-tG was decreased when cells were pre-treated with the receptor-specific antagonist α-BTX. However, pre-treatment did not block binding of rRABV-tG significantly, showing that RABV-G binds to other receptors on human MDMs as well. Alternative receptors may include additional nAChr, given that the expression of a wide array of nAChr (including α1, α-3-6, α9-10, β1-β4) have been described for human macrophages and macrophage-like cell lines (35, 36).

While major immune evasive mechanisms have been identified for the RABV nucleoprotein (N) and phosphoprotein (P), affecting RIG-I activation (12–14) and the IFN signal transduction pathway (15–17) or cytokine signaling (37) respectively, related mechanisms for the surface protein RABV-G have not yet been identified. Our results show that exposure of human MDMs to RABV leads to a decreased TNF-α response upon LPS challenge, caused by cytoplasmic retention of NF-κB. TNF-α levels and NF-κB nuclear translocation was completely restored when MDMs were pre-treated with the nAChr α7-specific antagonist α-BTX, showing that the observed anti-inflammatory effects of RABV were caused by binding of the RABV-G to nAChr α7. While multiple molecules have been found to induce the CAP in macrophages, including GTS-21 (38), CAP55 (39) and PNU-282987 (40), to our knowledge this is the first study showing that a viral protein is able to induce this pathway. The CAP might have additional effects on RABV pathogenesis and disease development, as was shown that the nAChr α7 antagonist GTS-21 attenuates the cytokine response in monocytes after stimulation with ligands for Toll-Like Receptor 2 (TLR2), TLR3, TLR4, TLR9, and RAGE. Furthermore, downregulating the inflammatory response was also observed in microglia (41), which are resident CNS macrophages that also express nAChr-α7 (42).

Given the importance of the NF-κB signaling pathway in the initiation of an immune response, multiple viruses [including Borna disease virus, Epstein-Barr virus, Hantaan virus, Hepatitis C virus, Poliovirus, Varicella-zoster virus and West Nile virus, as extensively reviewed (43)] have acquired mechanisms to inhibit NF-κB activation. We showed that RABV induced cytoplasmic retention of NF-κB *in vitro*, which is described as an essential step in activation of the CAP (44). For RABV it is known that the matrix (M) protein is able to inhibit RelAP43 activation, a splice variant of the NF-κB subunit RelA, leading to decreased expression of various innate immune genes (45–47). Similar effects have not yet been described for the other RABV proteins and our study is the first to show that RABV-G is able to specifically inhibit activation of the NF-κB signaling pathway. Contrary to our findings, microglia-like cell lines infected with the attenuated strain CVS-11 showed a strong activation of NF-κB (48). However, productive infection was observed in the cell lines used in that study.

Macrophages are APCs and potent cytokine producing cells, and therefore suppressed macrophage functioning can lead to decreased T cell activation. T cell activation requires three signals: T cell receptor (TCR) binding to antigens presented on major histocompatibility complexes (MHC) of APCs (signal 1), binding of costimulatory molecules (signal 2), and activation by cytokines. It has been proposed that IL-1 can provide the third signal for CD4^+^ cells (49, 50), resulting in T cell activation. Given that the inhibition of IL-1 had been described after induction of the CAP in macrophages (51, 52) the observed decrease in T cell proliferation might be caused by a decreased activation of CD4^+^ T cells. As a consequence, decreased cytokine production by CD4^+^ cells, and especially IL-2, can be a probable explanation for the significant decrease in CD8^+^ T cell proliferation. In our experiments we found a slight but non-significant decrease of 25% decrease in IL-2 production when T cells were co-cultured with RABV. Increased IL-10 production is associated with suppressive/anti-inflammatory or regulatory Th2 or Treg biased immune responses, and are known to decrease T cell proliferation and overall cytokine production (53–55). Induction of IL-10 is a known immunosuppressive strategy for viruses, and multiple viruses including human, equine and koi herpesviruses and poxvirus orf virus have functional IL-10 paralogs (56–59). Although RABV does not have an IL-10 paralog, it might affect IL-10 production through mechanisms that are yet to be revealed. While our results hint at macrophage-mediated T-cell suppression by RABV, more research is required to gain fuller insight into the functioning of these T cells.

Viral antigens can be sensed by macrophages and are capable of steering macrophage polarization in a certain direction (20, 21); the classically activated M1, or pro-inflammatory macrophage, and the M2 macrophage, also known as alternatively activated or anti-inflammatory macrophages (18, 19). Generally, macrophages polarize toward an M1 phenotype after recognition of intracellular pathogens, including viruses. We showed that exposure of human MDMs to a street RABV strain for 2 days significantly upregulated the M2-c marker CD163, while the typical M1-markers CD80 and HLA-DR remained unchanged. While more in-depth investigation on surface marker expression and macrophage functioning is necessary, the results hint at the ability of street RABV to shift macrophage polarization toward an M2-c phenotype. Studies on the effects of street RABV strains on polarization of primary macrophages are lacking, but studies using attenuated viruses CVS-11 and HEP show an opposite effect. Upregulated gene expression of iNOS and nitric oxide (NO) expression, a key characteristic of M1 macrophages, in the murine macrophage-like cell like RAW264 (23), and the attenuated SPBN-GAS was able to shift the polarization pattern of tumor-associated macrophages (TAMs), that normally resemble a M2-phenotype, toward an M1-phenotype in glioma-bearing mice (60).

CD163 is a scavenger receptor within the cysteine-rich family and is a marker of M2-c anti-inflammatory macrophages. Increased CD163 expression on macrophages has been reported during viral hepatitis (61), and infection of pigs with porcine reproductive and respiratory syndrome virus (62) and African swine fever virus (63). Furthermore, an increase in CD163^+^ macrophages was found in individuals infected with human immunodeficiency virus type 1 (64), as well as in rhesus macaques infected with simian immunodeficiency virus (65). Interestingly, the latter study showed a negative correlation between CD163^+^ macrophages and inflammatory infiltration in areas infected, indicating that CD163^+^ macrophages serve an anti-inflammatory or immunosuppressive role. In addition, CD163^+^ TAMs were found to suppress T cell proliferation in several disease models (66–69). Analysis of cytokines did not show a significant production of typical M1 or M2 cytokines by the RABV-stimulated macrophages, indicating that additional functional assays are required to fully investigate the effect of wildtype RABV on macrophage polarization.

In summary, our results show for the first time that a viral protein, the RABV-G of a street RABV strain, is able to induce the CAP in human MDMs. We show that this anti-inflammatory pathway is induced by binding of RABV-G to nAChr α7, using the specific antagonist α-BTX, leading to cytoplasmic retention of NF-κB. Besides the decreased inflammatory response upon challenge with LPS we found that exposing human MDMs to RABV leads to suppression of T cell proliferation *in vitro*, as well as an increase in the IL-10 production. We also show that exposure of human MDMs to RABV does not induce a typical M1 or M2 phenotype, based on surface marker expression and cytokine production. However, a significant upregulation of CD163, marker of a M2-c anti-inflammatory phenotype was observed. Given that the absence of a strong local innate immune response is beneficial for the virus, polarizing resident and infiltrating immune cells toward an anti-inflammatory state might be another mechanism of RABV to evade the immune system.

Future studies should focus on further understanding of the observed lack of adaptive immune response. Additional *in vitro* experiments in which the effects of “RABV-polarized” macrophages on T and B cells are investigated will allow evaluation of the role of macrophage suppression on the lack of neutralizing antibodies in RABV patients. *In vivo* experiments using targeted transgenic mice will allow reveal the role of macrophage suppression on the complete course of disease. Altogether, thorough insights into the different mechanisms that RABV uses to suppress the immune system are essential for the development of new and improved PEP and treatment options in RABV infection.

## Data Availability Statement

The raw data supporting the conclusions of this article will be made available by the authors, without undue reservation.

## Author Contributions

CE, LB, TK, and CG: conceptualization and methodology. CE: investigation. CE and CV: analysis. CV and CE: software. BM: contributed materials/reagents. CE, LB, TK, and CG: writing—original draft. CE, LB, CV, BM, MK, TK, and CG: writing—review and editing. All authors contributed to the article and approved the submitted version.

## Conflict of Interest

The authors declare that the research was conducted in the absence of any commercial or financial relationships that could be construed as a potential conflict of interest.
